# Comparison of Two Internet-Based Interventions for Problem Drinkers: Randomized Controlled Trial

**DOI:** 10.2196/jmir.2090

**Published:** 2012-08-01

**Authors:** John Alastair Cunningham

**Affiliations:** ^1^Social and Epidemiological ResearchCentre for Addiction and Mental HealthToronto, ONCanada

**Keywords:** Randomized controlled trial, problem drinking, alcohol abuse, Internet-based intervention, eHealth, brief intervention

## Abstract

**Background:**

Alcohol problems are a serious public health concern, and few problem drinkers ever seek treatment. The Internet is one means of promoting access to care, but more research is needed to test the best types of interventions to employ. Evaluation of Internet-based interventions that contain a variety of research-validated cognitive-behavioral tools, which have been shown to be helpful to those with more severe alcohol concerns, should be a priority.

**Objective:**

To evaluate whether providing access to an extended Internet intervention for alcohol problems offers additional benefits in promoting reductions in alcohol consumption compared with a brief Internet intervention. The hypothesis for the current trial was that respondents who were provided with access to an extended Internet intervention (the Alcohol Help Center [AHC]) would display significantly improved drinking outcomes at 6-month follow-up, compared with respondents who were provided with access to a brief Internet intervention (the Check Your Drinking [CYD] screener).

**Methods:**

A single-blinded randomized controlled trial with a 6-month follow-up. A general population sample of problem drinkers was recruited through newspaper advertisements in a large metropolitan city. Baseline and follow-up data were collected by postal mail.

**Results:**

A volunteer sample of problem drinkers of legal drinking age with home access to the Internet were recruited for the trial. Of 239 potential respondents recruited in 2010, 170 met inclusion criteria (average age 45 years; 101/170, 59.4% male; average Alcohol Use Disorders Identification Test [AUDIT] score of 22). Follow-up rates were 90.0% (153/170) with no adverse effects of the interventions reported. A repeated-measures multivariate analysis of variance of the outcome measures using an intent-to-treat approach found a significantly greater reduction in amount of drinking among participants provided access to the AHC than among participants provided access to the CYD (*P *= .046).

**Conclusions:**

The provision of the AHC gave additional benefit in the short term to problem drinkers over that seen from the research-validated CYD, indicating the benefits of promoting access to these interventions as one means of helping people with problem drinking concerns.

**Trial Registration:**

ClinicalTrials.gov NCT01114919; http://clinicaltrials.gov/ct2/show/NCT01114919 (Archived by WebCite at http://www.webcitation.org/68t1dCkRZ)

## Introduction

Alcohol is the third-leading cause of preventable death [1]. Unfortunately, the majority of people with drinking problems will never seek specialized addictions treatment [2]. Brief interventions in primary care settings have been identified as one means of addressing this important health problem [3]. However, given the prevalence of drinking problems and the resource restrictions in primary care settings, there is a need to also find alternative means of helping those with drinking problems.

Internet-based interventions have been identified as one promising option. Several reviews have concluded that there is a fast-developing evidence base for the efficacy of these interventions [4-8], particularly among college students, where the majority of these trials have been conducted. The evaluation of the efficacy of Internet-based interventions in general population samples is important if these brief interventions are to be promoted as helpful to anyone other than problem drinking, young adult college students. In addition, the majority of these Internet-based interventions have consisted of brief, personalized feedback interventions, which are thought to be useful to those with less severe alcohol problems. Evaluation of extended interventions that contain a variety of research-validated cognitive-behavioral tools, which have been shown to be helpful to those with more severe alcohol concerns, should also be a priority.

In this paper we report results of a randomized controlled trial testing the added benefit of providing access to such an extended Internet-based intervention, which contained an extensive array of cognitive-behavioral tools for problem drinkers (the Alcohol Help Center [AHC]), over the provision of a brief, personalized feedback Internet-based intervention (the Check Your Drinking [CYD] screener). Both of these Internet-based interventions are available free of charge on the Internet, making evaluations of their use of immediate benefit to problem drinkers. In addition, the CYD has already been subjected to four randomized controlled trials, in which the provision of this brief intervention yielded reductions in alcohol consumption among participants in a variety of different settings [9-12]. In the one study that employed a general population sample of problem drinkers [9], being provided access to the CYD resulted in an average reduction of 6 drinks at 3- and 6-month follow-up as compared with a no-intervention control group. Thus, the CYD brief intervention is an excellent comparator to evaluate whether providing access to an extended intervention (the AHC) would have additional benefit in promoting reductions in alcohol consumption. Finally, as there are few freely accessible, extended Internet-based interventions available [13,14] and as the evaluation of the efficacy of these extended Internet-based interventions has yielded mixed results [15], it is important to conduct further research in this area. The hypothesis for the current trial was that respondents in the extended Internet intervention condition (the AHC) would display significantly improved drinking outcomes at 6-month follow-up, compared with respondents in the brief Internet intervention condition (the CYD).

## Methods

Participants were recruited through newspaper advertisements in metropolitan Toronto, Canada (May to September 2010) asking for current drinkers interested in helping “in the process of developing and evaluating Internet-based interventions for alcohol users.” Interested potential participants called the telephone number provided and left their name and address to be sent a consent form and a baseline questionnaire by postal mail. The questionnaire contained a graphic describing a standard drink (note that a standard drink in Canada contains 13.6 g of alcohol).

Those returning the signed consent form and the completed baseline questionnaire were randomly assigned into one of two conditions: to be provided access to the brief Internet-based CYD brief personalized feedback intervention or to be provided access to the extended Internet-based AHC. Access was provided by sending each participant a unique password to be entered into a study-specific online portal (password sent in a letter by postal mail). Random numbers where generated in blocks of two using an online random numbers generator (Research Randomizer, Social Psychology Network; http://www.randomizer.org/) by the author. The random numbers list was used sequentially by a research assistant, who sent out the relevant intervention letter to participants in the order that their consent forms were returned. Participants were blind to the different experimental conditions in the study. Participants were followed up at 6 months and were compensated Can $20 for completing the follow-up questionnaire (questionnaire and check sent by postal mail). Follow-ups were conducted from November 2010 to June 2011. If participants did not return their original follow-up questionnaire within 1 month, they were sent a second questionnaire by postal mail. The research assistant in charge of follow-up was not blind to experimental condition, as the follow-up questionnaires were slightly different (outcome variables were identical and were asked about first, but some questions about impressions of the websites were different at the end of the questionnaire). The trial was approved by the standing research ethics committee for the Centre for Addiction and Mental Health.

**Figure 1:**
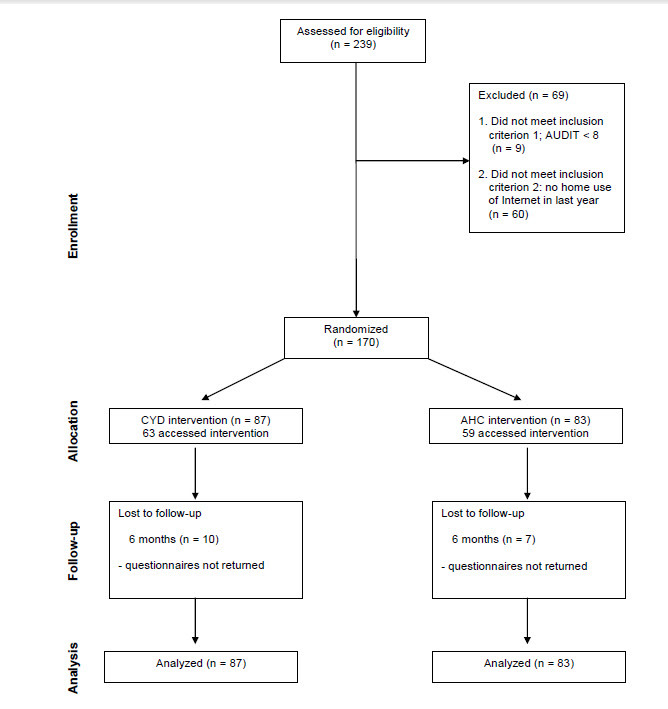
CONSORT diagram of participant recruitment. AHC = Alcohol Help Center, AUDIT = Alcohol Use Disorders Identification Test-C, CYD = Check Your Drinking.

As per protocol, the primary outcome measures were number of drinks consumed in a typical week, highest number of drinks on one occasion in the last 6 months, frequency of drinking, typical quantity of drinking on one occasion, and frequency of drinking 5 or more drinks on one occasion (the questions were the same for male and female participants). These last 3 items were combined to form the Alcohol Use Disorders Identification Test-Consumption subscale (AUDIT-C) [16]. For participants who did not return the follow-up questionnaire, missing data on the outcome measures were replaced with their respective baseline values. In addition, variables were examined for distributional properties and were Winsorized to deal with outliers. Exclusion criteria were being less than 19 years old (the legal drinking age in Ontario, Canada), having an AUDIT-C score of less than 8 (a score of 8 or more indicates current problem drinking) [17,18], and not having home access to the Internet. Lack of home Internet access was included in an attempt to include only regular Internet users in the trial. See Figure 1 for a CONSORT diagram of the trial.

### Statistical Analyses and Power Analysis

Results from a related self-help trial [19] were used to estimate that the addition of the extended intervention to the brief intervention would explain 3% more of the variance at 6 months. An increase of 3% of the explained variance is a medium effect size and corresponds to a mean decrease of approximately 3 drinks per week. Following the convention that studies should be designed to have a statistical power of at least 80%, and that hypotheses be tested at the .05 level of significance, SamplePower 1.0 [20] was used to estimate the required sample size. These specifications result in a final sample (required after attrition) of N = 170 (85 respondents per condition). Analyses were conducted using a repeated-measures multivariate analysis of variance with intervention condition (CYD vs AHC) as the between-participants variable and time (baseline vs 6 months) as the within-participants variable (including all outcome variables: typical weekly drinking, highest amount on one occasion, AUDIT-C score).

### Interventions

#### CYD Brief Intervention

The CYD screener (CheckYourDrinking.net) is a brief and personalized 18-item screener that has been designed to assess quantity and frequency of drinking, and the severity of drinking problems [21]. Following completion of the 18-item screener, the user is provided with a personalized final report that compares the person’s drinking with that of others in the general population of the same age, sex, and country of origin (for Canada, the United States, and the United Kingdom). The CYD is intended for quick completion in a single session, although participants could access it as often as they wanted.

#### AHC Extended Internet-Based Intervention

The AHC (AlcoholHelpCenter.net) is a website that has been developed to contain the cognitive-behavioral, motivational, and relapse prevention components that have been found to be effective in well-validated self-help books and other brief interventions. Specific sources for the content include (1) components found in the self-help book *DrinkWise *[22], (2) exercises from the self-help booklet *Alcohol and You*, developed by the principal investigator of this study [23], and (3) common relapse prevention exercises used in brief treatment modalities [24]. The main component of the AHC is divided into three sections: getting started (10 exercises focused on initiating change), dealing with difficulties (6 exercises covering some of the key issues that often occur as a problem drinker works on change), and maintenance (4 exercises designed to help participants maintain their change). In addition, there is a series of interactive tools that are useful throughout the change process, such as a drinking diary, where the participant is encouraged to track his or her drinking, and a blood alcohol calculator. Further, there are several elements that provide additional support to the participant: (1) an online support group that is moderated by health educators [25], (2) an email messaging system that provides the participant with encouragement and tips to deal with drinking concerns, and (3) a text messaging program for interested participants to be sent tips on how to deal with drinking problems. In summary, the AHC is a well-designed Internet-based intervention that contains many research-validated elements that have been shown in self-help books and other brief interventions to help problem drinkers. Participants have the option of completing whatever exercises they choose in whatever order they like. The AHC is intended for repeated use over an extended time period.

## Results

A total of 170 participants met inclusion criteria for this trial. Bivariate comparisons compared baseline demographic and drinking characteristics between experimental conditions (CYD, n = 87; AHC, n = 83) and found no significant differences between them (*P *> .05). Participants’ mean age was 45.2 (SD 12.2), 59.4% (101/170) were male, 58.8% (100/170) had some postsecondary education, 39% (67/170) were married or living with a partner, 55% (94/170) were employed full- or part-time, and more than half (92/170, 54%) had a yearly household income of Can $30,000 or more. Baseline drinking was heavy, with participants reporting an average AUDIT score of 22.1 (SD 7.6), typical weekly consumption of 31.7 (SD 18.4) drinks, and highest number of drinks consumed on one occasion of 13.7 (SD 7.2). A total of 71.8% (122/170) of participants used the password and logged in to their respective online intervention; however, all participants were included in the analysis in an intent-to-treat approach. Follow-up rates were good, with 90% (n = 153) completing the 6-month follow-up. There were no significant differences (*P *> .05) between experimental conditions, demographic characteristics, or drinking characteristics in the proportion of participants who completed the 6-month follow-up.

A multivariate analysis of variance was conducted and found a main effect for time (baseline vs 6 months; *F*_3,163 _= 15.0, *P *< .001, partial eta squared = 0.22). In addition, there was a significant interaction between time and condition (*F*_3,163 _= 2.7, *P *= .046, partial eta squared = 0.05). Further univariate tests found that the time × condition interaction was not significant for the number of drinks in a typical week variable (*F*_1,165 _= 0.4, *P *= .53), approached significance for the AUDIT-C variable (*F*_1,165 _= 3.6, *P *= .06, partial eta squared = 0.021), and was significant for the highest number of drinks on one occasion variable (*F*_1,165 _= 5.7, *P *= .02, partial eta squared = 0.034). Table 1 displays the means for the three variables at baseline and 6-month follow-up by condition. Participants in the AHC condition reduced the highest amount they consumed on one occasion from baseline to 6-month follow-up more than did participants in the CYD condition.

**Table 1:** Mean (SD) alcohol consumption and AUDIT-C^a ^scores at baseline and 6-month follow-up by intervention condition.

Variable	Baseline		6-month follow-up	
	Check Your Drinking (n = 87)	Alcohol Help Center (n = 83)	Check Your Drinking (n = 87)	Alcohol Help Center (n = 83)
No. drinks in a typical week	30.8 (19.2)	32.6 (17.9)	25.9 (18.7)	26.3 (20.7)
AUDIT-C score^b^	8.6 (2.0)	9.0 (1.5)	8.1 (2.5)	8.0 (2.6)
Highest number of drinks on one occasion^c^	13.1 (7.0)	13.9 (7.3)	11.5 (6.1)	10.3 (5.4)

^a ^Alcohol Use Disorders Identification Test-Consumption subscale is a composite measure that consists of respondents’ scores on frequency of drinking, drinks per drinking day, and frequency of 5 or more drinks on one occasion. Scores range from 0 to 12.

^b ^Time × condition, *P *= .06.

^c ^Time × condition, *P *= .02.

## Discussion

The current trial found a small but significant additional reduction in drinking among participants who were provided with access to the extended Internet-based intervention (the AHC) as compared with participants receiving the brief Internet-based intervention (the CYD) at a 6-month follow-up. The difference specifically had to do with reductions in amount of alcohol consumed during the participants’ heaviest drinking occasion. Given the increased risks associated with heavy drinking situations [26], this reduction is encouraging, although levels of consumption were still high in both conditions at follow-up. These results are also strengthened by the excellent follow-up rate in this trial (90%), something that has been put forth as a specific challenge in the conduct of eHealth intervention research [27]. The high follow-up rate could have resulted from the Can $20 honorarium or because much of the communication associated with the trial was conducted by postal mail rather than through email and online surveys. In addition, there was a trend (*P *= .06) toward an additional benefit of the AHC over the CYD on the AUDIT-C scores, a scale that is a composite measure of hazardous drinking. Finally, participants in both groups reduced their drinking in a typical week substantially (by 4.9 drinks per week in the CYD condition and 6.3 drinks per week in the AHC condition). These reductions are similar in scope to that seen in an earlier randomized controlled trial comparing the CYD with a no-intervention control [9]. However, there was no significant difference between the CYD and the AHC conditions in amount of drinking in a typical week in the current trial, so no claims regarding the efficacy of the AHC in reducing typical weekly drinking can be made based on these results (ie, there is no evidence that these results could not have been due to regression to the mean).

A strength, and a simultaneous limitation, of this trial was the severity of current drinking problems in the participants (an AUDIT score of 20 or more is taken as a reasonable proxy of alcohol dependence, and the mean AUDIT score in the current trial was 22) [17]. Thus, while it is encouraging to see an additional impact of the AHC over the CYD among this sample of people with relatively severe alcohol consumption, realistically, this may not be the ideal target population for an Internet-based intervention (even an extended one) [17,28]. Hybrid versions of Internet-mediated interventions where therapists interact with clients through an Internet portal is a promising alternative for treating those with more severe alcohol problems if face-to-face options do not exist [29,30]. Future research trials evaluating this intervention, or other extended Internet-based interventions of a similar nature, would be well served to use recruitment methods that emphasize those with hazardous, but not severe, alcohol problems. Other limitations that could be addressed in a systematic replication of this trial are a lack of a no-interventions control group (which would allow us to make statements about the efficacy of both Internet-based interventions to reduce typical weekly consumption), data reporting on the degree of engagement with the AHC, and a long-term follow-up that would allow statements to be made about any extended impact of these interventions. Finally, the results of this trial relied on self-report of the participants. While there is good evidence that self-reports of drinking are generally reliable in this type of trial [31] and, more important, there is no reason to expect that there would be differences between experimental conditions in the accuracy of participants’ self-reports, further replications of this research would benefit from validation of participants’ self-reports.

Despite these limitations, the results of this trial are important, as they demonstrate the added benefit of an extended Internet-based intervention over a brief, personalized feedback intervention for problem drinkers. Systematic replications of these findings would allow the development of an adequate research base supporting this highly accessible and cost-effective means of providing assistance to problem drinkers who might otherwise receive no help for this important health concern.

## Abbreviations

AHC: Alcohol Help Center
AUDIT: Alcohol Use Disorders Identification Test
AUDIT-C: Alcohol Use Disorders Identification Test-Consumption subscale
CYD: Check Your Drinking

## Multimedia Appendix 1

CONSORT-ehealth V.1.6.1 checklist [32].
