# Supplementary material for: Comparison of Two Internet-Based Interventions for Problem Drinkers: Randomized Controlled Trial
Source: J Med Internet Res. 2012 Aug 1;14(4):e107. doi: 10.2196/jmir.2090 (PMC3799611; doi:10.2196/jmir.2090)
Supplement: Supplementary file 1 [file jmir_v14i4e107_app1.pdf]

CONSORT-EHEALTH Checklist V1.6 Report	Manuscript Number	2090
Date completed	8/25/2011 14:50:29	
by	John Cunningham	
Randomized controlled trial of two Internet-based interventions for problem drinkers		
TITLE		
**1a-i) Identify the mode of delivery in the title**		
"Internet-based interventions"		
**1a-ii) Non-web-based components or important co-interventions in title**		
There were no other important co-interventions or non-web-based components		
**1a-iii) Primary condition or target group in the title**		
"for problem drinkers"		
ABSTRACT		
**1b-i) Key features/functionalities/components of the intervention and comparator in the METHODS section of the ABSTRACT**		
"A single-blinded randomized controlled trial with a six-month follow-up." - main feature/component of the trial		
**1b-ii) Level of human involvement in the METHODS section of the ABSTRACT**		
**1b-iii) Open vs. closed, web-based (self-assessment) vs. face-to-face assessments in the METHODS section of the ABSTRACT**		
"A general population sample of problem drinkers was recruited through newspaper advertisements in a large metropolitan city." - addresses how participants were recruited		
**1b-iv) RESULTS section in abstract must contain use data**		
"A volunteer sample of problem drinkers of legal drinking age with home access to the Internet were recruited for the trial. Of 239 potential respondents recruited in 2010, 170 met inclusion criteria.Follow-up rates were 90% with no adverse effects of the interventions reported"		
**1b-v) CONCLUSIONS/DISCUSSION in abstract for negative trials**		
INTRODUCTION		
**2a-i) Problem and the type of system/solution**		
"given the prevalence of drinking problems and the resource restrictions in primary care settings, there is a need to also find alternate means of helping those with drinking problems."<br><br>"IBIs (Internet-based interventions) have been identified as one promising option and several reviews have concluded that there is a fast developing evidence base for the efficacy of these interventions [4-8], particularly among college students where the majority of these trials have been conducted. The evaluation of the efficacy of IBIs in general population samples is of importance if these brief interventions are to be promoted as helpful to anyone other than problem drinking, young adult, college students. In addition, the majority of these IBIs have consisted of brief, personalized feedback interventions, which are thought to be useful to those with less severe alcohol problems. Evaluation of extended interventions that contain a variety of research validated, cognitive-behavioral tools, which have been shown to be helpful to those with more severe alcohol concerns, should also be a priority."		
**2a-ii) Scientific background, rationale: What is known about the (type of) system**		
Both of these IBIs are available, free-of-charge on the Internet, making evaluations of their use of immediate benefit to problem drinkers. In addition, the CYD has already been subjected to four randomized controlled trials in which the provision of this brief intervention has yielded reductions in alcohol consumption among participants in a variety of different settings [9-12]. Thus, the CYD brief intervention is an excellent comparator to evaluate whether providing access to an extended intervention (the AHC) would have additional benefit in promoting reductions in alcohol consumption. Finally, as there are few, freely accessible, extended IBIs available [13,14] and as the evaluation of the efficacy of these extended IBIs has yielded mixed results [15], it is important to conduct further research in this area.		
METHODS		
**3a) CONSORT**		
The hypothesis for the current trial was that respondents in the extended Internet intervention condition (the AHC) would display significantly improved drinking outcomes at six-month follow-up, compared to respondents in the brief Internet intervention condition (the CYD).		
**3b-i) Bug fixes, Downtimes, Content Changes**		
**4a-i) Computer / Internet literacy**		
Participants were excluded if did not have home access to the Internet. This was an attempt to only include regular Internet users in the trial.		
**4a-ii) Open vs. closed, web-based vs. face-to-face assessments:**		
"Participants were recruited using newspaper advertisements in metropolitan Toronto (May to September, 2010) asking for current drinkers interested in helping "in the process of developing and evaluating Internet-based interventions for alcohol users." Interested potential participants called the telephone number provided and left their name and address to be sent a consent form and a baseline questionnaire. Those returning the signed consent form and completed baseline questionnaire were randomized into one of two conditions: to be provided access to the brief Internet-based Check Your Drinking brief personalized feedback intervention or to be provided access to the extended Internet-based Alcohol Help Center. Access was provided by sending each participant a unique password to be entered into a study specific online portal."		
**4a-iii) Information giving during recruitment**		
**4b-i) Report if outcomes were (self-)assessed through online questionnaires**		
"primary outcome measures were number of drinks consumed in a typical week, highest number of drinks on one occasion in the last 6 months, frequency of drinking, typical quantity of drinking on one occasion, and frequency of drinking five of more drinks on one occasion" - these measures were assessed through questionnaires that were part of each Internet-based intervention.		
**4b-ii) Report how institutional affiliations are displayed**		
**5-i) Mention names, credential, affiliations of the developers, sponsors, and owners**		
**5-ii) Describe the history/development process**		
**5-iii) Revisions and updating**		
**5-iv) Quality assurance methods**		
**5-v) Ensure replicability by publishing the source code, and/or providing screenshots/screen-capture video, and/or providing flowcharts of the algorithms used**		
**5-vi) Digital preservation**		
**5-vii) Access**		
Check Your Drinking Brief Intervention: CheckYourDrinking.net<br><br>Alcohol Help Centre Extended Internet-Based Intervention: AlcoholHelpCentre.net<br><br>"Access was provided by sending each participant a unique password to be entered into a study specific online portal." Access is free of charge.<br><br>editors/reviewers/readers can access both websites for demonstration purposes without the need of a specific password or authorization		
**5-viii) Mode of delivery, features/functionalities/components of the intervention and comparator, and the theoretical framework**		

"Check Your Drinking Brief Intervention: The Check Your Drinking screener (CYD; CheckYourDrinking.net) is a brief and personalized 18 item screener that has been designed to assess quantity and frequency of drinking, and the severity of drinking problems [21]. Following the completion of the 18 item screener, the user is provided with a personalized Final Report that compares the person’s drinking with that of others in the general population of the same age, sex and country of origin (for Canada, the U.S.A., and the U.K).

Alcohol Help Centre Extended Internet-Based Intervention: The Alcohol Help Centre (AHC; AlcoholHelpCentre.net) is a website that has been developed to contain the cognitive-behavioral, motivational, and relapse prevention components that have been found to be effective in well validated self-help books and other brief interventions. Specific sources for the content include: 1) components found in the self-help book, Drinkwise [22]; 2) exercises from the self-help booklet, Alcohol and You, developed by the PI of this paper [23]; and 3) common relapse prevention exercises used in brief treatment modalities [24]. The main components of the AHC are divided into three sections; getting started (10 exercises focused on initiating change), dealing with difficulties (6 exercises covering some of the key issues that often occur as a problem drinker works on change), and maintenance (4 exercises designed to help participants maintain their change). In addition, there are a series of interactive tools that are useful throughout the change process, such as a drinking diary where the participant is encouraged to track their drinking, and a blood alcohol calculator. Further, there are several elements that provide additional support to the participant: 1) a support group that is moderated by health educators [25]; 2) an e-mail messaging system that provides the participant with encouragement and tips to deal with drinking concerns; and 3) a text messaging program for interested participants to be sent tips on how to deal with drinking problems. In summary, the AHC is a well-designed Internet-based intervention that contains many research validated elements that have been shown to help problem drinkers in self-help books and other brief interventions."

5-ix) Describe use parameters

5-x) Clarify the level of human involvement

5-xi) Report any prompts/reminders used

There were no prompts or reminders used in this trial.

5-xii) Describe any co-interventions (incl. training/support)

There were no co-interventions as part of this trial.

6a-i) Online questionnaires: describe if they were validated for online use and apply CHERRIES items to describe how the questionnaires were designed/deployed

6a-ii) Describe whether and how “use” (including intensity of use/dosage) was defined/measured/monitored

6a-iii) Describe whether, how, and when qualitative feedback from participants was obtained

7a-i) Describe whether and how expected attrition was taken into account when calculating the sample size

7b) CONSORT

"Following the convention that studies should be designed to have a statistical power of at least 80%, and that hypotheses be tested at the .05 level of significance, SamplePower 1.0 [20] was used to estimate the required sample size. These specifications result in a final sample (required after attrition) of N = 170 (85 respondents per condition)."

8a) CONSORT

"Random numbers where generated in blocks of two using an online random numbers generator (Research Randomizer) by the first author. The random numbers list was used sequentially by a research assistant who send out the relevant intervention letter to participants in the order that their consent forms were returned."

8b) CONSORT

"Random numbers where generated in blocks of two using an online random numbers generator (Research Randomizer) by the first author."

9) CONSORT

"Random numbers where generated in blocks of two using an online random numbers generator (Research Randomizer) by the first author. The random numbers list was used sequentially by a research assistant who send out the relevant intervention letter to participants in the order that their consent forms were returned. Participants were blind to the different experimental conditions in the study."

10) CONSORT

"Random numbers where generated in blocks of two using an online random numbers generator (Research Randomizer) by the first author. The random numbers list was used sequentially by a research assistant who send out the relevant intervention letter to participants in the order that their consent forms were returned."

11a-i) Specify who was blinded, and who wasn’t

"Participants were blind to the different experimental conditions in the study."

11a-ii) Discuss e.g., whether participants knew which intervention was the “intervention of interest” and which one was the “comparator”

11b) CONSORT

Yes. Similarities and differences of the two interventions can be gleaned from the Interventions subsection of the paper.

12a) CONSORT

"Analyses were conducted using a repeated measure MANOVA (Multivariate Analysis of Variance) with intervention condition (CYD versus AHC) as the between subjects variable and time (baseline versus six-months) as the within subjects variable (including the all outcome variables: typical weekly drinking, highest amount on one occasion, AUDIT-C)."

12a-i) Imputation techniques to deal with attrition / missing values

"Missing data on the outcome measures were replaced with their respective baseline values. In addition, variables were examined for distributional properties and were Winsorized to deal with outliers."

12b) CONSORT

There are no other, additional analyses reported.

RESULTS

13a) CONSORT

"A total of 170 participants met inclusion criteria for this trial. Bivariate comparisons compared baseline demographic and drinking characteristics between experimental condition (CYD, n = 87; AHC, n =83) and found no significant differences between condition (P > .05)."

13b) CONSORT

Loss of participants after randomization is addressed in Figure 1. consort diagram. Participants were lost at follow-up because questionnaires were not returned. "A total of 72% of participants used the password and logged into their respective online intervention, however, all participants were included in the analysis in an intent to treat approach. Follow-up rates were good, with 90% (n = 153) completing the six-month follow-up."

13b-i) Attrition diagram

Depicted in Figure 1.

14a) CONSORT

"Participants were recruited using newspaper advertisements in metropolitan Toronto (May to September, 2010)" "Follow-ups were conducted from November 2010 to June of 2011"

14a-i) Indicate if critical “secular events” fell into the study period

14b) CONSORT

The trial was not terminated early. Recruitment concluded once sample size specifications were reached.

15) CONSORT

Clinical characteristics are provided in Table 1.

15-i) Report demographics associated with digital divide issues

"Particpants' mean (SD) age was 45.2 (12.2), 59% were male, 59% had some post-secondary education, 39% lived were married or living with a partner, 55% were full or part-time employed, and more than half (54%) had a yearly household income of CAN$30,000 or more. Baseline drinking was heavy, with participants reporting an average (SD) AUDIT score of 22.1 (7.6), typical weekly consumption of 31.7 (18.4), and highest number of drinks consumed on one occasion of 13.7 (7.2)."		
**16-i) Report multiple "denominators" and provide definitions**		
"Bivariate comparisons compared baseline demographic and drinking characteristics between experimental condition (CYD, n = 87; AHC, n =83)"		
**16-ii) Primary analysis should be intent-to-treat**		
"all participants were included in the analysis in an intent to treat approach."		
**17a) CONSORT**		
"A MANOVA was conducted and found a main effect for Time (baseline versus six-months), F (3, 163) = 15.0, P < .001, Partial Eta Squared = .22. In addition, there was a significant interaction between Time and Condition, F (3, 163) = 2.7, P = .046, Partial Eta Squared = .05. Further univariate tests found that the Time by Condition interaction was not significant for the variable, number of drinks in a typical week, F(1, 165) = 0.4, p = .53, was marginally significant for the AUDIT-C variable, F(1, 165) = 3.6, P = .06, Partial Eta Squared = .021, and was significant for the highest number of drinks on one occasion variable, F(1, 165) = 5.7, P = .02, Partial Eta Squared = .034. Table 1 displays the means for the three variables at baseline and six-months follow-up by condition. Participants in the AHC condition reduced the highest amount they consumed on one occasion from baseline to six-month follow-up more than participants in the CYD condition."		
**17a-i) Presentation of process outcomes such as metrics of use and intensity of use**		
**17b) CONSORT**		
"A MANOVA was conducted and found a main effect for Time (baseline versus six-months), F (3, 163) = 15.0, P < .001, Partial Eta Squared = .22. In addition, there was a significant interaction between Time and Condition, F (3, 163) = 2.7, P = .046, Partial Eta Squared = .05. Further univariate tests found that the Time by Condition interaction was not significant for the variable, number of drinks in a typical week, F(1, 165) = 0.4, p = .53, was marginally significant for the AUDIT-C variable, F(1, 165) = 3.6, P = .06, Partial Eta Squared = .021, and was significant for the highest number of drinks on one occasion variable, F(1, 165) = 5.7, P = .02, Partial Eta Squared = .034. Table 1 displays the means for the three variables at baseline and six-months follow-up by condition. Participants in the AHC condition reduced the highest amount they consumed on one occasion from baseline to six-month follow-up more than participants in the CYD condition."		
**18) CONSORT**		
"Further univariate tests found that the Time by Condition interaction was not significant for the variable, number of drinks in a typical week, F(1, 165) = 0.4, p = .53, was marginally significant for the AUDIT-C variable, F(1, 165) = 3.6, P = .06, Partial Eta Squared = .021, and was significant for the highest number of drinks on one occasion variable, F(1, 165) = 5.7, P = .02, Partial Eta Squared = .034."		
**18-i) Subgroup analysis of comparing only users**		
**19) CONSORT**		
There were no harms or unintended effects in each group.		
**19-i) Include privacy breaches, technical problems**		
**19-ii) Include qualitative feedback from participants or observations from staff/researchers**		
DISCUSSION		
**20-i) Typical limitations in ehealth trials**		
"A strength, and a simultaneous limitation of this trial was the severity of current drinking problems in the participants (an AUDIT score of 20 or more is taken as a reasonable proxy of alcohol dependence and the mean AUDIT score in the current trial was 22). Thus, while it is encouraging to see an additional impact of the AHC over the CYD among this sample of people with relatively severe alcohol consumption, realistically, this is not the ideal target population for an Internet-based intervention (even an extended one)."<br><br>"Other limitations that could be addressed in a systematic replication of this trial were a lack of a no interventions control group (which would allow us to make statements about the efficacy of both IBIs to reduce typical weekly consumption), and a long-term follow-up that would allow statements about any extended impact of these interventions. Finally, the results of this trial relied on self-report of the participants."		
**21-i) Generalizability to other populations**		
**21-ii) Discuss if there were elements in the RCT that would be different in a routine application setting**		
**22-i) Restate study questions and summarize the answers suggested by the data, starting with primary outcomes and process outcomes (use)**		
The current trial found a small but significant additional reduction in drinking among participants provided access to the extended IBI (the AHC) as compared to participants receiving the brief IBI (the CYD) at a six-month follow-up. The difference specifically had to do with reductions in amount of alcohol consumed during the participants' heaviest drinking occasion. Given the increased risks associated with heavy drinking situations [26], this reduction is encouraging, although levels of consumption were still high in both conditions at follow-up. These results are also strengthened by the excellent follow-up rate in this trial (90%), something that has been put forth as a specific challenge in the conduct of eHealth intervention research [27]. In addition, there was a trend (P = .06) towards an additional benefit of the AHC over the CYD on the AUDIT-C scores, a scale that is a composite measure of hazardous drinking. Finally, participants in both groups reduced their drinking in a typical week substantially (4.9 drinks per week in the CYD condition and 6.3 drinks per week in the AHC condition). These reductions are similar in scope to that seen in an earlier randomized controlled trial comparing the CYD to a no intervention control [9]. However, there was no significant difference between the CYD versus the AHC conditions in amount of drinking in a typical week in the current trial so no claims regarding the efficacy of the AHC in reducing typical weekly drinking can be made based on these results.		
**22-ii) Highlight unanswered new questions, suggest future research**		
"Future research trials evaluating this intervention, or other extended IBIs of a similar nature, would be well served to use recruitment methods that emphasized those with hazardous, but not severe alcohol problems."		
Other information		
**23) CONSORT**		
"Trial Registration: ClinicalTrials.gov: NCT01114919"		
**24) CONSORT**		
Full trial protocol is not available for access		
**25) CONSORT**		
"Support to Centre for Addiction and Mental Health for salary of scientists and infrastructure has been provided by the Ontario Ministry of Health and Long Term Care."		
**X26-i) Comment on ethics committee approval**		
"The trial was approved by the standing research ethics committee for the Centre for Addictions and Mental Health."		
**x26-ii) Outline informed consent procedures**		
**X26-iii) Safety and security procedures**		
**X27-i) State the relation of the study team towards the system being evaluated**		
"The author has no conflicts of interest to declare."		
